# Intranasal peptide-induced tolerance and linked suppression: consequences of complement deficiency

**DOI:** 10.1111/imm.12358

**Published:** 2014-12-08

**Authors:** Liliane Fossati-Jimack, Guang Sheng Ling, Lucie Baudino, Marta Szajna, Kiruthika Manivannan, Jade Chen Zhao, Robert Midgley, Jian-Guo Chai, Elizabeth Simpson, Marina Botto, Diane Scott

**Affiliations:** 1 Centre for Complement and Inflammation Research, Imperial College LondonLondon, UK; 2 Molecular Immunology, Imperial College LondonLondon, UK

**Keywords:** complement, dendritic cells, regulatory T cells, rodent, tolerance

## Abstract

A role for complement, particularly the classical pathway, in the regulation of immune responses is well documented. Deficiencies in C1q or C4 predispose to autoimmunity, while deficiency in C3 affects the suppression of contact sensitization and generation of oral tolerance. Complement components including C3 have been shown to be required for both B-cell and T-cell priming. The mechanisms whereby complement can mediate these diverse regulatory effects are poorly understood. Our previous work, using the mouse minor histocompatibility (HY) model of skin graft rejection, showed that both C1q and C3 were required for the induction of tolerance following intranasal peptide administration. By comparing tolerance induction in wild-type C57BL/6 and C1q-, C3-, C4- and C5-deficient C57BL/6 female mice, we show here that the classical pathway components including C3 are required for tolerance induction, whereas C5 plays no role. C3-deficient mice failed to generate a functional regulatory T (Treg) –dendritic cell (DC) tolerogenic loop required for tolerance induction. This was related to the inability of C3-deficient DC to up-regulate the arginine-consuming enzyme, inducible nitric oxide synthase (*Nos-*2), in the presence of antigen-specific Treg cells and peptide, leading to reduced Treg cell generation. Our findings demonstrate that the classical pathway and C3 play a critical role in the peptide-mediated induction of tolerance to HY by modulating DC function.

## Introduction

The complement system, a major component of innate immunity, also has an important regulatory role in adaptive immune responses, where it is involved in both the induction and control of B-cell and T-cell activation.^1^,^2^

A role for the classical pathway in immune regulation stems from the observations that, in humans, deficiencies in C1q or C4 predispose to systemic lupus erythematosus.^3^ The absence of these components causes impaired clearance of apoptotic cells^4^ and defective B-cell regulation.^5^,^6^ C3 has also been reported to be important in the regulation of immune responses, particularly in the suppression of contact sensitization and generation of oral tolerance,^7^–^9^ and has been recently demonstrated to have an immunosuppressive role in the differentiation of myeloid suppressor cells.^10^ Conversely, complement, particularly C3, is required for optimal B-cell immunity^11^–^13^ and T-cell priming.^2^,^14^,^15^ Signalling by the complement fragments C3a and C5a through their receptors, C3aR and C5aR, has been implicated in T-cell activation directly^16^,^17^ or indirectly through dendritic cell (DC) activation.^18^–^20^ The mechanisms dictating the modulatory function of complement in adaptive immunity remain poorly understood and depend on the experimental conditions applied.

Similarly the role of complement in tolerance remains unresolved. Although there is evidence that C3 is needed for the rejection of fully MHC mis-matched kidney grafts,^21^ we and others have found, using the minor histocompatibility (HY) model of skin graft rejection, that C1q and C3 were required not for rejection, but for the induction of tolerance.^23^ We previously showed that intranasally (i.n.) administered MHC class II-restricted HY-A^b^*Dby* peptide led to its presentation by immature DC and the generation of peptide-specific CD4 regulatory T (Treg) cells.^24^,^25^ Following skin grafting, or immunization with male cells, the effect of these Treg cells was to impair the presentation of the full cohort of MHC class I and II male epitopes resulting in a diminished cytotoxic T lymphocyte response and graft tolerance.^24^ These findings were consistent with the notion of a regulatory feedback loop between Treg cells and DC and generation of a population of tolerogenic DC.^26^,^27^

Here, we have extended our observations and uncovered some potential mechanisms by which C3 might regulate the induction of peptide-induced tolerance and linked suppression.

## Materials and methods

### 

#### Mice

C57BL/6 (B6) were from Harlan (Bicester, UK). B6.*C1qa*^*–/–*^,^28^ B6.*C3*^*–/–*^,^29^ B6.*C4*^*–/–*^29 and B6.*C5*^*–/–*^30 were previously described and backcrossed onto C57BL/6 for 10 generations. Mice with T-cell receptors transgenic for the HY-A^b^*Dby* peptide (Marilyn mice)^31^ were provided by Dr O. Lantz (Paris, France). All experiments on animals complied with standard conditions and were covered by a UK Home Office Project License.

#### Tolerance induction and skin grafting

The HY-A^b^*Dby* peptide (100 μg diluted in 20 μl PBS) (NAGFNSNRANSSRSS; ThinkPeptides, Sarasota, FL) was administered i.n. on 3 consecutive days to female mice anaesthetized under isoflurane. Control mice received PBS. Ten days later, skin grafting was performed^32^ using male and female tail skin grafted onto the lateral thorax of syngeneic female recipients. Grafts were scored as rejected when < 10% viable tissue remained. After 100 days, selected mice were injected intraperitoneally with 5 × 10^6^ male splenocytes and 7 days later the HY-specific CD8 T-cell response was measured using HY-Db*Uty* dextramer (Immudex, Copenhagen, Denmark).

#### Isolation of splenic DC and T-cell proliferation

Splenic DC were isolated using a CD11c-positive selection kit (Miltenyi Biotec GmbH, Bergisch Gladbach, Germany). Then, 2·5 × 10^4^ CD4^+^ Marilyn T cells were added to purified DC and incubated for 72 hr. Wells were pulsed with 0·5 μCi [^3^H]thymidine for the last 18 hr. Plates were counted using a Wallac Trilux 1450 MicroBeta liquid scintillation counter (PerkinElmer LAS, Seer Green, UK).

#### Adoptive cell transfer

Marilyn CD4^+^ CD25^−^ T cells were isolated from Marilyn mice using a CD4 negative selection kit (Miltenyi Biotec GmbH), followed by a CD25 selection kit (Miltenyi). Cells were labelled with 5 μm carboxyfluorescein diacetatesuccinimidyl ester (CFSE; Molecular Probes®, Life Technologies, Paisley, UK) according to the manufacturer's instructions. Then 2 × 10^6^ to 5 × 10^6^ labelled cells were injected intravenously into each recipient. Results are shown as proliferation index, calculated using flowjo software.

#### Flow cytometry

Cells were stained using standard protocols in the presence of saturating anti-Fc*γ*RII/III (2.4G2). The following antibodies were used: anti-mouse CD90.1 (OX.7), anti-mouse CD25 (PC61), anti-mouse CD4 (GK1.5), anti-mouse CD40 (3/23), anti-mouse CD86 (GL1), anti-mouse H-2A (2G9) from BD Biosciences (San Diego, CA); anti-rat/mouse forkhead box p3 (Foxp3; FJK-16s), anti-mouse CD8 (53–6.7) from Life Technologies. Data were acquired using a FACSVerse (Becton-Dickinson, Mountain View, CA) and analysed using flowjo software, (TreeStar, Ashland, OR). Foxp3 staining was performed according to the manufacturer (eBioscience, San Diego, CA).

#### Treg cell generation and suppression assay

CD4^+^ cells were isolated by negative selection (Miltenyi Biotec GmbH) and cultured in RPM1-1640 at 10^6^ cells/ml in U-bottom wells coated with anti-CD3 antibody (1 μg/ml, 1452C11, Biolegend, San Diego, CA) and anti-CD28 antibody (1 μg/ml; eBioscience); in the presence of recombinant human transforming growth factor-*β* (TGF-*β*; 10 ng/ml, PeproTech, Rocky Hill, NJ), anti-interferon-*γ* antibody (2·5 μg/ml, Biolegend) and r-mouse interleukin-2 (5 ng/ml) for 4 days. The T-cell suppression assay was performed as previously described.^33^ Proliferation was expressed as a division index, calculated using flowjo software.

#### Co-culture assays

Bone marrow dendritic cells (BMDC) were generated as previously described.^34^ Marilyn induced Treg (iTreg) cells were obtained from naive *Rag2*^*–/–*^ Marilyn spleen cells (5 × 10^5^ cells/ml) cultured with BMDC (10^5^ cells/ml), HY-A^b^*Dby* peptide (10 nm), recombinant human TGF-*β* (2 ng/ml) and retinoic acid (1 μm, Sigma, St Louis, MO) for 5 days. Live cells were recovered on a gradient density (Lympholyte, Cedarlane Laboratories, Burlington, ON, Canada). Staining indicated ≥ 70% Foxp-3^+^ cells. Fresh BMDC (10^6^ cells/ml) were co-cultured with iTreg cells (10^6^ cells/ml) in the presence or absence of HY-A^b^*Dby* peptide (1 nm) for 48 hr.

#### Quantitative real-time RT-PCR

RNA was extracted from cell cultures using RNeasy columns (Qiagen, Hilden, Germany). The cDNA was generated using an iScript cDNA synthesis Kit (BioRad, Hercules, CA). Real-time PCR were performed using specific primers (see Supporting information, Table S1) with SYBR green master mix (Applied Biosystems®, Life Technologies, Paisley, UK) and analysed on a ViiA™7 RealTime PCR system (Applied Biosystem®, Life Technologies). Relative quantities were calculated using the ΔΔCt method.^35^ Samples that gave no detectable signal were attributed a Ct value of 40; samples were normalized to *Gapdh*.

#### Statistical analysis

Comparisons between two groups were performed using two-tailed unpaired Student's *t*-test. Graft survival was compared using the log-rank test. Statistical significance is defined as *P* ≤ 0·05.

## Results

### Early components of the classical pathway are required for induction of nasal tolerance

**Figure 1:**
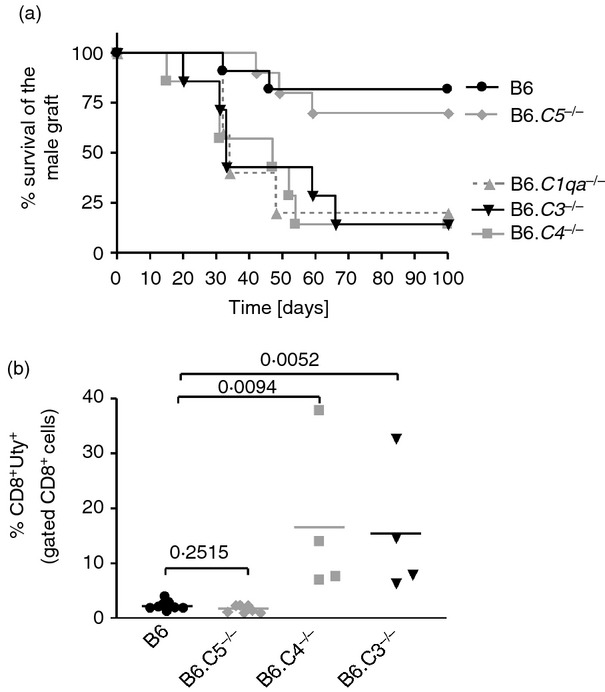
Impaired induction of tolerance in mice lacking early components of the classical complement pathway. B6 wild-type (WT; black circle), B6.*C1qa*^–/–^*-* (grey triangles), B6.*C4*^–/–^ (grey squares), B6.*C3*^–/–^ (black triangles) and B6.*C5*^–/–^ (grey diamonds) female mice were given HY-A^b^*Dby* peptide (100 μg for 3 days) intranasally. (a) Survival of syngeneic male skin grafts transplanted on peptide-treated female WT (*n* = 11), B6.*C1qa*^–/–^ [*n* = 5, median survival time (MST) 34 days, *P* = 0·0141], B6.*C4*^–/–^ (*n* = 7, MST 47 days, *P* = 0·0045), B6.*C3*^–/–^ (*n* = 7, MST 33 days, *P* = 0·0030), B6.*C5*^–/–^ (*n* = 10, *P* = 0·6039). Syngeneic female grafts were accepted indefinitely. Statistical analysis was performed using the log-rank method comparing separately each complement-deficient strain to peptide-treated WT B6. (b) 100 days after skin grafting, selected mice were injected intraperitoneally with 5 × 10^6^ male splenocytes and 7 days later spleen cell suspensions were analysed for HY-specific CD8 T-cell responses (HY-Db*Uty* dextramer^+^ CD8 T cells) by flow cytometry. B6 (*n* = 9), B6.*C5*^–/–^ (*n* = 7), B6.*C4*^–/–^ (*n* = 4), B6.*C3*^–/–^ (*n* = 4). Statistical analysis: *t*-test.

C57BL/6 (B6) wild-type (WT) female mice can be tolerized to the male HY antigen by i.n. administration of the HY-A^b^*Dby* peptide,^25^ but not in the absence of C1q or C3.^22^ To establish whether the terminal pathway of complement was also required, C5-deficient (B6.*C5*^–/–^) female mice received i.n. HY-A^b^*Dby* peptide and 10 days later were grafted with male skin. Peptide-treated B6.*C5*^–/–^ females became tolerant, maintaining the male graft as well as WT mice (Fig. 1a). In contrast, as previously reported,^22^ female mice lacking C1q (B6.*C1qa*^–/–^) or C3 (B6.*C3*^–/–^) started to lose their grafts with median survival times (MST) of 34 and 37 days, respectively and fewer than 20% retained their graft after 100 days. Syngeneic female grafts were accepted indefinitely. As both C1q^36^,^37^ and C3^7^–^10^ have been shown to independently regulate immune responses, we performed similar experiments with C4-deficient female mice (B6.*C4*^–/–^) to investigate whether the early components of the classical pathway were required for tolerance induction. No long-term tolerance was achieved in these mice; they rejected their grafts with an MST of 47 days, which was not statistically different from that of B6.*C1qa*^–/–^ and B6.*C3*^–/–^ mice (Fig. 1a). Moreover, these graft survival times were not different from that of non-peptide treated WT mice (MST 31 days). These results therefore indicate a role for the early phase of the classical pathway of complement activation, rather than an independent role for the individual components C1q and C3 or the terminal pathway. To further confirm these findings, some mice were given an *in vivo* boost with male cells 100 days after grafting and their CD8 T-cell response was measured 7 days later using an HY-peptide-specific (HY-Db*Uty*) dextramer (Fig. 1b). The tolerant WT and B6.*C5*^–/–^ mice gave a very low anti-HY-specific CD8 T-cell response, whereas the non-tolerant C3-deficient and C4-deficient mice showed large expansions of the HY-Db*Uty* dextramer^+^ CD8 T-cell population, indicating a failure in the non-tolerant animals to control the cytotoxic response against HY. Hence, the early phase of the classical pathway plays a key role in this model of tolerance. As C3 is central to this, we elected to focus on this molecule to investigate possible mechanisms.

### C3 does not affect peptide dissemination *in vivo*

**Figure 2:**
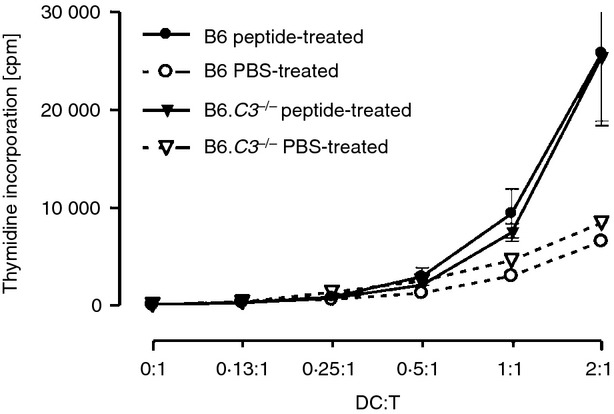
C3 deficiency does not alter the distribution of the peptide. B6 wild-type (WT; *n* = 3) and B6.*C3*^–/–^ (*n* = 3) female mice were given the HY-A^b^*Dby* peptide twice (100 μg at time 0 and 6 hr later) intranasally, control mice received PBS. Twenty-four hours after the first peptide dose, splenic dendritic cells (DC) were isolated and cultured with Marilyn T cells (25 000 T cells/well) at the ratios indicated. Proliferation was assessed by pulsing with [^3^H]thymidine for the final 18 hr of the 72 hr incubation. Data are shown as mean ± SEM, Student's *t-*test. (DC : T ratio 2 : 1 *P* = 0·9704, 1 : 1 *P* = 0·5030).

The initial phase in this model of tolerance induction is the presentation of the peptide by antigen-presenting cells, most likely DC. Twenty-four hours after the i.n. administration the peptide is found in CD11c-positive DC in spleen, and in peripheral and cervical draining lymph nodes.^24^ To determine whether complement affected the peptide dissemination and/or presentation, B6 WT and B6.*C3*^–/–^ female mice were given i.n. HY-A^b^*Dby* peptide. Twenty-four hours later splenic DC were isolated and used to stimulate HY-A^b^*Dby*-specific CD4^+^ T cells from TCR transgenic Marilyn mice (Marilyn T cells). The purity, percentage of CD8^+^/CD8^−^ DC and expression levels of CD40, CD86 and H2-A^b^ were similar (data not shown). Regardless of their complement status splenic, DC from the peptide-treated mice were equally able to stimulate the proliferation of the Marilyn T cells (Fig. 2); hence complement does not influence the distribution or initial presentation of the peptide.

### The early phase of tolerance induction is defective in B6.C3^–/–^ mice

**Figure 3:**
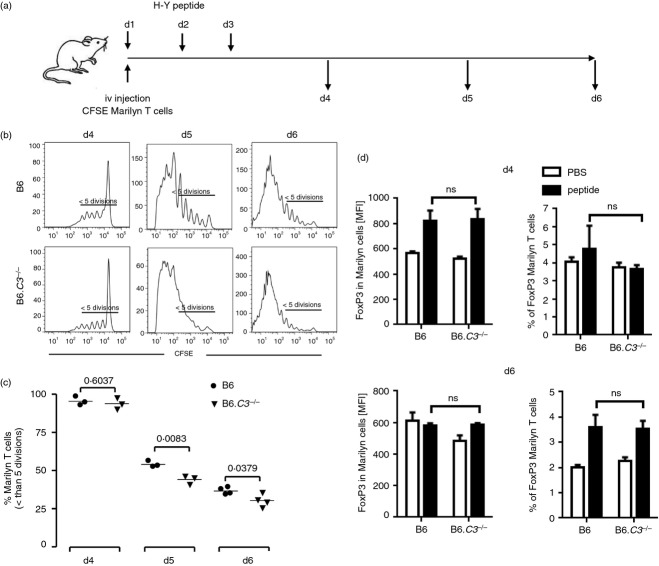
Increased antigen-specific T-cell proliferation in response to intranasal (i.n.) peptide delivery in B6.*C3*^–/–^ mice. 2 × 10^6^ CFSE-labelled Marilyn CD90.1 CD4^+^ CD25^−^ T cells were injected intravenously into female recipient B6 wild-type (WT) or B6.*C3*^–/–^ mice (three or four animals per group). Mice received i.n. three doses of the HY-A^b^*Dby* peptide. Splenocytes were recovered 4, 5 and 6 days after the first peptide dose and proliferation of Marilyn T cells (gated as CD4^+^, CD90.1^+^) was analysed by flow cytometry. (a) Experimental schedule. (b) Representative data of gated Marilyn T-cell proliferation. (c) Percentage of Marilyn T cells with less than five divisions. (d) Foxp3 expression [mean fluorescence intensity (MFI)] in Marilyn T cells (left panel) and percentage of Foxp3^+^ Marilyn T cells (right panel). Results from control mice receiving PBS are indicated by open columns; those from peptide-treated mice are shown by filled columns. Data are shown as mean ± SEM. Statistical analysis by unpaired *t*-test; *P*-values as indicated; ns, non-significant.

To study the early phase of tolerance induction, concomitantly with i.n. peptide administration, we adoptively transferred intravenously CFSE-labelled Marilyn CD4 T cells to act as indicators of the endogenous anti-HY response. This protocol has been previously shown to have no effect on peptide-mediated tolerance induction.^24^ Proliferation was assessed at days 4, 5 and 6 (Fig. 3a).

No differences in proliferation were observed at day 4, confirming that complement does not play a role in the early distribution and presentation of the peptide. By day 5 Marilyn T cells transferred into B6.*C3*^–/–^ mice had divided extensively, and by day 6 most of the cells were CFSE negative. In contrast, Marilyn T cells transferred into WT mice demonstrated a significantly slower rate of proliferation (Fig. 3b,c).

To determine whether complement affected Treg cell induction in the adoptively transferred Marilyn T cells, we examined Foxp3 expression at days 4 and 6 (Fig. 3d). At day 4 the mean fluorescence intensity of Foxp3 in the Marilyn T cells was increased compared with the PBS-treated controls,^38^ irrespective of the complement status of the recipient mice (Fig. 3d, left hand panel). The percentage of Foxp3^+^ Marilyn T cells was similar. By day 6, the percentage of Foxp3^+^ Marilyn T cells had increased in the peptide-treated compared with the PBS-treated mice, again regardless of the complement status of the hosts (Fig. 3d, right hand panel). Hence, although C3 was required for the peptide-mediated regulation of proliferation of the adoptively transferred Marilyn T cells, it appeared to have little effect on the induction of Foxp3^+^ Treg cells.

### The early phase of the induction of linked tolerance is impaired in B6.C3^–/–^ mice

**Figure 4:**
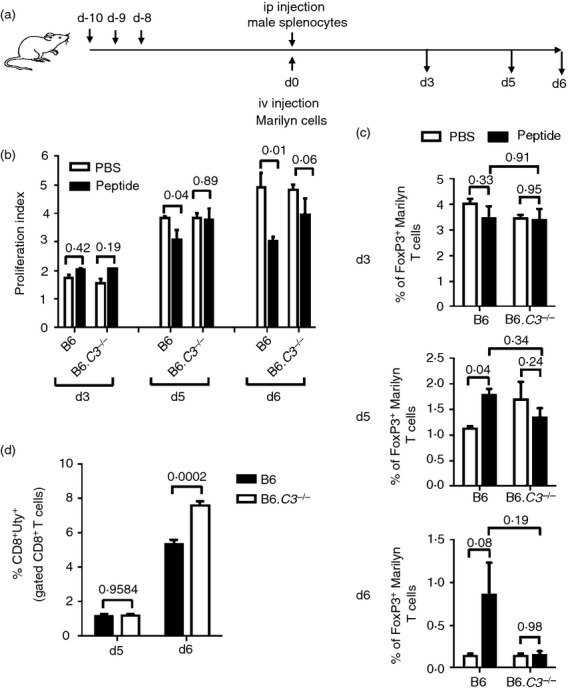
Induction of antigen-specific regulatory T (Treg) cells in peptide-treated mice after immunization with male cells. Ten days after intranasal (i.n.) treatment with three doses of peptide (100 μg), female B6 wild-type (WT) or B6.*C3*^–/–^ mice (three or four animals per group) were challenged intraperitoneally with 5 × 10^6^ male splenocytes. The antigen-specific CD4 T-cell response was monitored by infusing 2 × 10^6^ CFSE-labelled Marilyn CD90.1 CD4^+^ CD25^−^ T cells intravenously. Three, 5 and 6 days after the challenge, splenocytes were recovered and the proliferation of Marilyn T cells (gated as CD4^+^ CD90.1^+^) was analysed by flow cytometry (a) Experimental schedule. (b) Proliferation indices of Marilyn T cells. (c) Percentage of Foxp3^+^ Marilyn T cells. Results from mice treated with PBS (open column) and peptide (filled column) are shown. Bars indicate SEM; *P*-values of unpaired *t*-test are indicated. (d) Five and 6 days after the challenge, splenocytes were recovered and analysed for HY-specific CD8 T-cell responses (HY-Db*Uty* dextramer^+^ CD8 T cells) by flow cytometry. Bars indicate SEM. *P*-values of unpaired *t*-test are indicated.

To examine the early phase of induction of linked tolerance, to the additional HY epitopes expressed on male tissue, B6 WT and B6.*C3*^–/–^ mice were given an intraperitoneal injection of male splenocytes 10 days after receiving three doses of HYA^b^*Dby* peptide or control PBS. At the same time, CFSE-labelled Marilyn T cells were injected intravenously (Fig. 4a). At day 3 there was minimal proliferation by the Marilyn T cells, even from the peptide-treated mice. However, by days 5 and 6 a marked increase in the proliferation of Marilyn T cells in response to the immunization with male cells was found in the control PBS-treated mice regardless of their complement status as well as in the peptide-treated B6.*C3*^–/–^ mice. In contrast, there was significantly less proliferation in the peptide-treated WT mice compared with the PBS-treated controls. (Fig. 4b).

Endogenous Treg cells are expanded in peptide-treated mice following immunization with male antigen.^24^,^39^ We therefore examined the percentage of Foxp3^+^ cells within the adoptively transferred Marilyn T-cell population following encounter with male antigen. At day 3 there was no expansion of Foxp3^+^ cells. However, by days 5 and 6 the percentage of Foxp3^+^ Marilyn T cells was increased in the peptide-treated WT mice compared with PBS-treated controls, whereas in B6.*C3*^–/–^ mice no such induction was observed (Fig. 4c).

We also examined expansion of endogenous HY-specific CD8 effector T cells. On day 5 there was no expansion of HY-specific CD8 T cells within the CD8 population from either peptide-treated WT or B6.*C3*^–/–^ mice. However, by day 6 we observed expansions that were significantly greater in the B6.*C3*^–/–^ compared with WT animals (Fig. 4d).

Taken together, these findings indicate that in C3-deficient mice the generation of Treg cells is impaired, perhaps because of a defect in the induction of the tolerogenic loop. Consistent with this, both the adoptively transferred Marilyn T cells and the endogenous HY-specific CD8 T cells divided more extensively in the peptide-treated C3-deficient mice than in the corresponding WT controls.

### C3 deficiency in T cells does not affect their capacity to become Treg cells

Administration of the peptide to B6.*C3*^–/–^ mice resulted in a defective induction of Treg cells within the adoptively transferred C3-sufficient Marilyn T cells (Fig. 4c). However, it was not possible to evaluate the endogenous HY-peptide specific C3-deficient Treg cells *in vivo* or *ex vivo*. Therefore we assessed whether C3 deficiency could affect the *in vitro* induction of Treg cells from naive CD4 T cells. Using a standard protocol^40^ we found that the generation of iTreg cells from WT and B6.*C3*^–/–^ mice was similar (see Supporting information, Fig. S1A). In addition, both iTreg populations were functionally equivalent in suppression assays, irrespective of whether the responder T cells were WT (see Supporting information, Fig. S1B) or C3-deficient (Fig. S1C). These results suggest that there is no inherent inability in the B6.*C3*^–/–^ T cells to become Treg cells.

### Induction of tolerogenic DC appears to be impaired in C3-deficient mice

**Figure 5:**
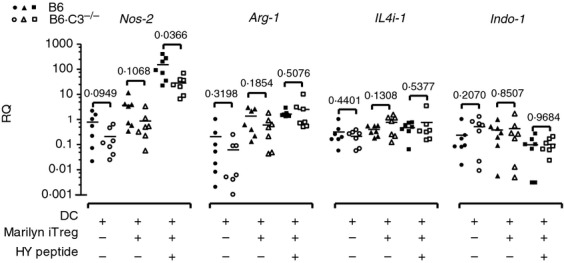
Induction of amino acid-consuming enzymes in dendritic cells (DC) by inducible regulatory T (iTreg) cells. Bone-marrow-derived dendritic cells (BMDC) from B6 wild-type (WT; closed symbols) or B6.*C3*^–/–^ mice (open symbols) were cultured either alone or with induced Marilyn Treg cells (ratio 1 : 1), in the absence or in the presence of 1 nm HY-A^b^*Dby* peptide for 48 hr. Samples were analysed for gene expression by real-time PCR using specific primers for *Nos-2*, *Arg-1*, *IL4i-1* and *Indo-1*. Relative expression was normalized to *Gapdh*. Data are represented as relative quantification (RQ); each dot represents a separate biological replicate (seven per group). *P*-values are indicated (*t*-test).

Although it is well established that DC make up a central component of the tolerance-inducing pathway, the identification and characterization of these cells *in vivo* have proven to be difficult. It has recently been reported that the generation of Treg cells from naive T cells, a process known as infectious tolerance, was a result of the induction of amino-acid-metabolizing enzymes in the antigen-presenting DC. In addition, culture of *in-vitro*-generated Treg cells with BMDC in the presence of cognate peptide induced a similar set of amino-acid-consuming enzymes, arginase 1 (*Arg-1*), nitric oxide synthase 2 (*Nos-2*), interleukin-4-induced 1 (*Il4i-1*) and indolamine 2,3-dioxygenase (*Indo-1*) in the DC.^41^ Using a similar approach, we cultured WT and B6.*C3*^–/–^ BMDC with Foxp3^+^ Marilyn iTreg cells. This induced C3 expression (see Supporting information, Fig. S2) and an increase in *Nos-2* transcription in the WT DC compared with DC alone. Transcription was further increased in the presence of cognate peptide (Fig. 5). When C3-deficient DC were used, the expression of the *Nos-2* gene in the presence of peptide was significantly lower (Fig. 5). *Arg-1* expression was up-regulated following incubation of the DC with Marilyn iTreg cells, but the presence of the cognate peptide did not further increase its expression, as was reported previously.^41^ However, there was no significant difference in expression of *Arg-1* between the WT and C3-deficient DC. We found no obvious differences in the expression of *IL-4i* or *Indo-1* in the co-culture of DC and Marilyn iTreg cells with or without peptide compared with DC alone. Again the absence of C3 did not alter the expression of these genes.

## Discussion

The role of complement in the regulation of immune responses remains controversial. Here we have extended our previous studies and show that, in addition to C1q and C3, C4 is also needed for peptide-induced tolerance, confirming the key role of the early components of the classical pathway. The finding that B6.*C5*^–/–^ mice can be tolerized as well as WT demonstrates that the terminal pathway is not involved.

We and others have shown that this model of peptide-induced tolerance involves the induction of a dominant regulatory mechanism mediated by Foxp3^+^ CD4 Treg cells and resulting in impaired expansions of HY-specific CD4 and CD8 T cells.^24^,^25^,^39^ The lack of tolerance in C3-deficient mice was accompanied by increased proliferation of the adoptively transferred HY-specific CD4 T cells and expansion of endogenous HY-specific CD8 T cells. This correlated with a lower frequency of Foxp3^+^ CD4 Treg cells within the adoptively transferred indicator Marilyn T cells. Collectively, these results suggest that the generation of Treg cells and the subsequent Treg–DC tolerogenic loop^26^,^27^ was defective in the absence of C3. Using naive CD4 T cells we were unable to demonstrate a role for C3 in the induction of Treg cells *in vitro*. Moreover, the cells from both WT and B6.*C3*^–/–^ animals were equally suppressive, demonstrating that in the presence of exogenous TGF-*β*, there is no intrinsic defect in the ability of B6.*C3*^–/–^ T cells to differentiate into functional Foxp3^+^ Treg cells. Our observations that there were fewer Foxp3^+^ Marilyn T cells, which are complement-sufficient, following adoptive transfer into peptide-treated B6.*C3*^–/–^ recipients immunized with male cells, pointed to a defect within the DC rather than the T-cell populations.

How does the classical pathway of complement affect tolerance induction? It has recently been shown that all the subcomponents of the C1 complex (C1q, C1r, C1s) are not only synthesized by, but are also present on the surface of human monocytes,^42^ supporting the idea of local complement activation via the classical pathway on the surface on monocytes and possibly other bone-marrow-derived cells. Although we found that both C1q and C4 were needed for tolerance induction, C3 deficiency gave the same phenotype, indicating that C3, with its associated regulatory function^7^–^9^ may play a central role in mediating tolerance in this model. Following the cognate interaction between DC and CD4 T cells, both cell types can produce C3, although T cells to a far less extent (17 and see Supporting information, Fig. S2). A possible scenario would therefore be that local production of complement causing the activation of C3 would lead to the generation of C3 activation fragments, C3a and C3b.

Despite evidence suggesting that C3a and C5a signalling through their respective C3aR and C5aR receptors is important for T-cell activation^16^,^17^,^19^,^20^ and that this signalling leads to suppression of Treg cell generation,^43^ our results and those of others^23^ clearly show that C3 is required for tolerance induction. Our observation that C5 deficiency did not affect peptide-induced tolerance also argues against a role for C5aR signalling. In addition, the C3-deficient mice used in our study cannot generate any intracellular C3a fragment as they lack the corresponding genetic region,^29^ making it unlikely that the effects we observed were mediated by intracellular or extracellular C3aR. Nonetheless, the possibility of a C3-mediated intracellular signalling mediated by other C3 fragments cannot be excluded.^44^

We have previously shown that following i.n. peptide administration, a population of Treg cells with up-regulated expression of Foxp3, CTLA4, programmed cell death 1 and lymphocyte activation gene 3 as well as interferon-*γ* and interleukin-13^24^ was generated. Expression of these molecules causes down-modulation of DC maturation and co-stimulatory capacity^45^,^46^ and the induction of the amino acid catabolizing enzymes indolamine 2,3-dioxygenase^47^,^48^ and Arginase 1.^49^ This gives rise to tolerogenic DC, which in turn interact with naive CD4 T cells recognizing cognate MHC peptide and causing down-regulation of the T-cell mammalian target of rapamycin (mTOR) signalling pathway, up-regulation of Foxp3 and further Treg cell generation.^50^,^51^ Hence establishing a tolerogenic loop.^26^,^27^

Related to this, here we have identified a potential explanation for the defective ability of the B6.*C3*^–/–^ DC to induce non-responsiveness: an impaired induction of *Nos2* expression following culture with *in vitro*-generated HY-specific Treg cells in the presence of the cognate peptide. *Nos2* encodes inducible nitric oxide synthase 2 (iNOS), an enzyme shown to have an important role in the reduction of available arginine, which is crucial for the induction of infectious tolerance through the mTOR signalling pathway.^41^,^50^ Although NO generation by iNOS has been associated with pro-inflammatory responses, there is now good evidence that NO has direct immunoregulatory effects.^52^ Given the importance of iNOS in immune regulation, it is plausible that C3 contributes to tolerance induction in the intranasal peptide model by modulating its expression.

In summary, our results have shown that the complement classical pathway, and particularly C3, is important for the induction of tolerance to HY by the intranasal administration of a single MHC class II-restricted HY peptide. The defect is likely to be related to the inability of complement-deficient mice to establish the Treg–DC tolerogenic loop required for the induction and maintenance of tolerance. We found no evidence of a defect in the ability of C3-deficient T cells to generate Treg cells using established *in vitro* protocols arguing against an intrinsic T-cell defect. Rather the finding that B6.*C3*^–/–^ BMDC produced significantly less iNOS following incubation with T cells and cognate antigen, suggests that the defect is at the level of the antigen-presenting cells. In the absence of C3, the induction of amino-acid-depleting enzymes in DC is impaired, causing reduced Treg cell generation, possibly as a result of impaired down-regulation of the mTOR pathway.

## Abbreviations

BMDC: bone-marrow-derived dendritic cells
B6: C57BL/6
CFSE: carboxyfluorescein diacetatesuccinimidyl ester
DC: dendritic cells
Foxp3: Forkhead box p3
i.n.: intranasal
iTreg: induced regulatory T
MST: median survival time
iNOS: nitric oxide-synthase-2
TGF-*β*: transforming growth factor-*β*
Treg: regulatory T
WT: wild-type

## Supporting Information

**Table S1.** Primer sequences for real-time RT-PCR. **Figure S1.** Induction of regulatory T cells *in vitro*. **Figure S2.** C3 expression in dendritic cell–T-cell co-culture conditions.
