# Supplementary material for: Intranasal peptide-induced tolerance and linked suppression: consequences of complement deficiency
Source: Immunology. 2014 Dec 8;144(1):149–57. doi: 10.1111/imm.12358 (PMC4264918; doi:10.1111/imm.12358)
Supplement: Supplementary file 1 [file imm0144-0149-sd1.pdf]

Supplementary table I: Primer sequences for real-time RT-PCR.

Gene	Forward primer	Reverse Primer
Nos-2	GGCAGCCTGTGAGACCTTTG	GCATTGGAAGTGAAGCGTTTC
Arg-1	GGAATCTGCATGGGCAACCTGTGT	AGGGTCTACGTCTCGCAAGCCA
IL-4i	AGGACATCTACCAGATGGCA	CATCTCCCAGGAGCTGCA
C3	CAAACAGATGTCCCTGACCA	TGCTTTTGAGTTTGGCATGA
Gapdh	TGTGTCCGTCGTGGATCTGA	TTGCTGTTGAAGTCGCAGGAG

For *Ido1* Mm01143935_g1 Taqman primer was used.

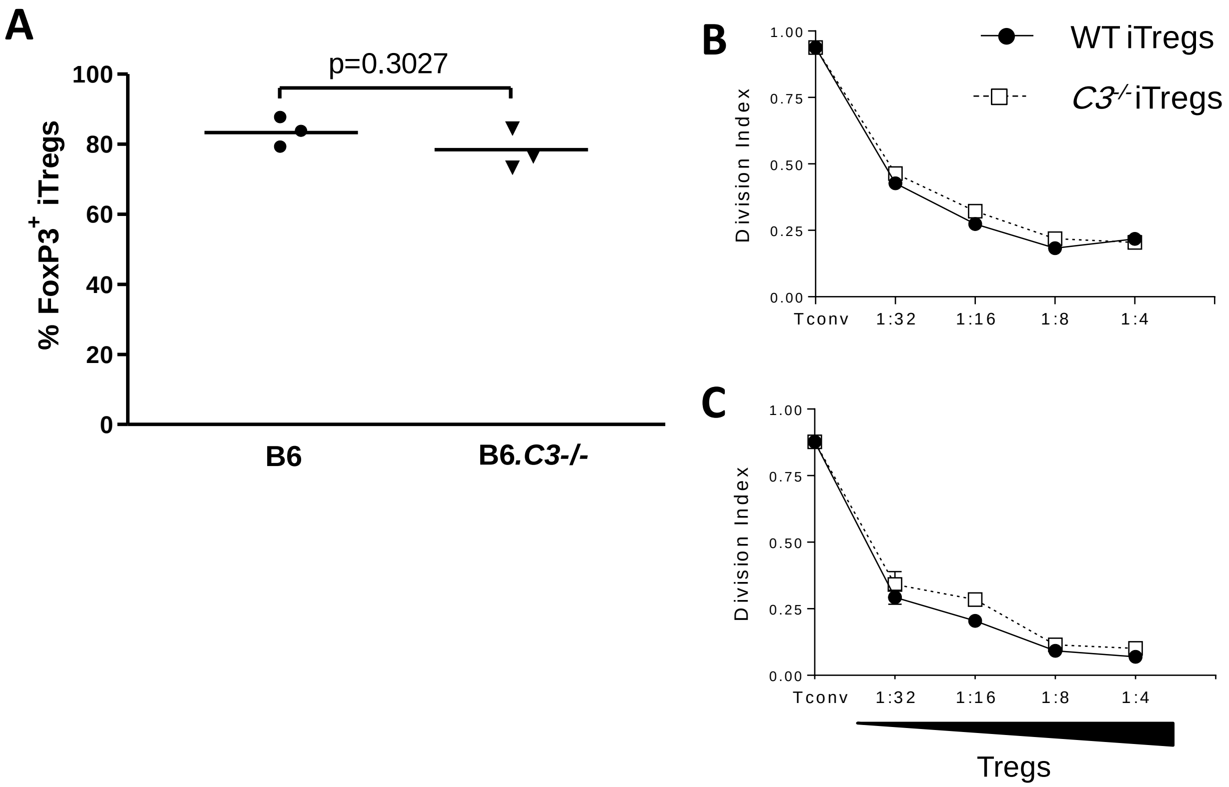

Supplementary Figure 1: Induction of Treg *in vitro*.

(A) Purified CD4 T cells from B6 WT (circles) or B6.*C3-/-* (triangles) mice (3 per group) were activated for 3 days with anti-CD3 and anti-CD28 beads, recombinant IL-2 and TGF-β in the presence of an anti-IFN-γ antibody. The percentage of $Foxp3^{+}$ $CD4^{+}$ T cells was analysed by flow cytometry. (B) and (C) Suppression assays with iTreg. CFSE-labelled $CD25^{-}CD4^{+}$ responder T cells from B6 WT (B) or B6.*C3-/-* (C) mice were stimulated with anti-CD3 and CD28 beads in the presence of increasing concentrations of iTregs from WT B6 (closed circles) or B6.*C3-/-* (open squares) mice. The division index of the responder cells was used to assess the proliferation. The data are representative of three independent experiments. Data are shown as mean ± SEM.

Supplementary Figure 1

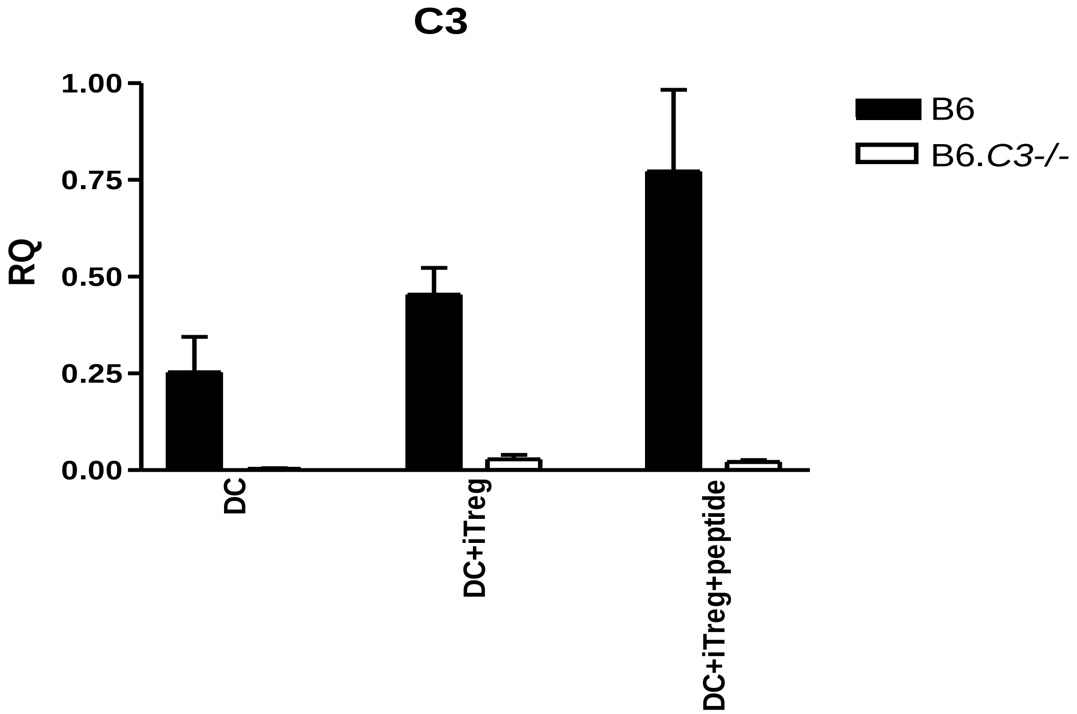

Supplementary Figure 2: C3 expression in DC-T cell co-culture conditions. BMDCs from B6 WT (filled columns) or C3-deficient mice (open columns) were cultured either alone or with induced Marilyn Treg (ratio 1:1), in the absence or in the presence of 1nM HY-A^b*Dby* peptide for 48 hours. Samples were then analysed for C3 expression by real-time PCR using specific primers for C3. (Supplementary Table I) Relative expressions were normalised to *Gapdh*. The low levels of C3 expression detected in C3-deficient conditions originated from the C3 sufficient Marilyn T cells. Data are represented as relative quantification (RQ), mean of 7 individual biological replicates ± SEM.

Supplementary Figure 2
